# A field-programmable gate array based on wafer-scale 2D semiconductor

**DOI:** 10.1093/nsr/nwaf458

**Published:** 2025-10-31

**Authors:** Qicheng Sun, Mingrui Ao, Xiangqi Dong, Kaichuang Shi, Saifei Gou, Yuxuan Zhu, Zhejia Zhang, Jinshu Zhang, Yan Hu, Zhengjie Sun, Xinyu Chen, Lingli Wang, Wenzhong Bao, Peng Zhou

**Affiliations:** State Key Laboratory of Integrated Chips and Systems, College of Integrated Circuits and Micro-Nano Electronics, Fudan University, Shanghai 200433, China; State Key Laboratory of Integrated Chips and Systems, College of Integrated Circuits and Micro-Nano Electronics, Fudan University, Shanghai 200433, China; State Key Laboratory of Integrated Chips and Systems, College of Integrated Circuits and Micro-Nano Electronics, Fudan University, Shanghai 200433, China; State Key Laboratory of Integrated Chips and Systems, College of Integrated Circuits and Micro-Nano Electronics, Fudan University, Shanghai 200433, China; State Key Laboratory of Integrated Chips and Systems, College of Integrated Circuits and Micro-Nano Electronics, Fudan University, Shanghai 200433, China; State Key Laboratory of Integrated Chips and Systems, College of Integrated Circuits and Micro-Nano Electronics, Fudan University, Shanghai 200433, China; State Key Laboratory of Integrated Chips and Systems, College of Integrated Circuits and Micro-Nano Electronics, Fudan University, Shanghai 200433, China; State Key Laboratory of Integrated Chips and Systems, College of Integrated Circuits and Micro-Nano Electronics, Fudan University, Shanghai 200433, China; State Key Laboratory of Integrated Chips and Systems, College of Integrated Circuits and Micro-Nano Electronics, Fudan University, Shanghai 200433, China; State Key Laboratory of Integrated Chips and Systems, College of Integrated Circuits and Micro-Nano Electronics, Fudan University, Shanghai 200433, China; State Key Laboratory of Integrated Chips and Systems, College of Integrated Circuits and Micro-Nano Electronics, Fudan University, Shanghai 200433, China; State Key Laboratory of Integrated Chips and Systems, College of Integrated Circuits and Micro-Nano Electronics, Fudan University, Shanghai 200433, China; State Key Laboratory of Integrated Chips and Systems, College of Integrated Circuits and Micro-Nano Electronics, Fudan University, Shanghai 200433, China; Shaoxin Laboratory, Shaoxing 312000, China; Shanghai AtomIC Technology, Shanghai 201318, China; State Key Laboratory of Integrated Chips and Systems, College of Integrated Circuits and Micro-Nano Electronics, Fudan University, Shanghai 200433, China; Shaoxin Laboratory, Shaoxing 312000, China

**Keywords:** 2D material, field-programmable gate array, integrated circuit, irradiation resistance

## Abstract

In recent years, 2D transition-metal dichalcogenides (2D-TMDs) have garnered significant attention owing to their unique electronic properties. Particularly in the application of short-channel field-effect transistors, 2D-TMDs have demonstrated notable superiority compared with silicon. This is primarily attributed to their exceptional low off-state current enabled by their monolayer structure, which is crucial for suppressing short-channel effects. Consequently, 2D-TMDs have been widely recognized as promising candidates for future technology. However, current 2D-TMD-based semiconductor devices are predominantly focused on simple logic circuits, lacking practical validation for large-scale functional circuits. In this work, we present a top-gate 2D field-programmable gate array (FPGA) based on the 2D-TMD material molybdenum disulfide. This circuit marks the first demonstration of an FPGA that was constructed from 2D-TMDs, comprising ∼4000 field effect transistors (FETs) and programmable. In addition, our circuits demonstrate unique advantages in irradiation resistance. Our work provides a significant impetus for the further development of 2D-TMD technology towards practical applications.

## Teaser

Researchers demonstrate the first field-programmable gate array built from wafer-scale 2D molybdenum disulfide semiconductors, containing ∼4000 transistors and showing high radiation resistance.

## INTRODUCTION

As modern integrated circuits approach atomic dimensions, growing challenges are faced in further transistor scaling. The complexity and low returns of advanced technologies such as the gate-all-around field effect transistor (GAA FET) and the complementary FET (CFET) are impeding Moore’s Law. In contrast, low-dimensional materials [1,2], such as 2D transition-metal dichalcogenides (2D-TMDs), with their atomically thin nature, offer promising alternative solutions by effectively suppressing short-channel effects and reducing the leakage current [1,3]. Due to the fact above, 2D-TMDs are considered as the next-generation semiconductor, which has garnered considerable attention from both academia and industry [4–7]. So far, many of the challenges previously encountered in fabricating 2D-TMD-based integrated circuits (ICs) have been progressively overcome. At the material level, methods for large-scale chemical vapor deposition (CVD) growth of high-quality 2D-TMDs have advanced, with labs producing 4- to 12-inch single-crystal molybdenum disulfide (MoS_2_) [8,9,10–16], laying a solid foundation for large-scale ICs. At the process level, research efforts are dedicated to single-step fabrication process optimization. This includes optimizing source/drain processes to reduce contact resistance and achieve ohmic contacts [17–20], optimizing dielectric layers to minimize the off-state current of FETs [18,21–23], modulating the threshold voltage of FETs via the dipole effect within the dielectric layer [24] and others. At the integration level, machine-learning methods are being applied [8] for holistic optimization and 3D integration [25,26] is being explored to further leverage the ultra-thin advantages of 2D-TMDs. Nevertheless, the circuit complexity of 2D-TMD-based processes remains relatively limited, typically confined to basic logic gates [11,12,27–29], with a few studies achieving complex circuits of ∼100 FETs [7,8,15,30–32].

2D-TMDs, while versatile, currently struggle to meet the performance, power efficiency and reliability requirements of customized application-specific ICs (ASICs). Field-programmable gate arrays (FPGAs), which are one kind of programmable logic, with their programmability and scalability, often replace ASICs in many scenarios, especially for rapid prototyping verification. Moreover, due to its relatively fixed circuit modules, the FPGA is suitable for the preparation of emerging 2D-TMDs in semiconductor technology, especially in the field of 2D material ICs. Unlike traditional ASICs, FPGAs, post-fabrication, can be configured and programmed by users to implement specific logic functions according to their requirements by using Hardware Description Languages such as Verilog or VHSIC Hardware Description Language. This inherent programmability and high flexibility have led to the widespread adoption of FPGAs in scenarios demanding high parallelism and customized hardware, including digital signal processing, embedded systems and network communication [33–35]. In recent years, FPGAs have also been utilized in acceleration tasks such as deep learning and neural network inference [36]. Utilizing 2D-TMDs to fabricate FPGAs presents numerous advantages. Emerging memory technologies based on 2D-TMDs are currently undergoing extensive research. Innovative device structures that leverage the unique properties of 2D-TMDs are promising for next-generation memory solutions. They hold the potential to overcome the memory wall bottleneck through compute-in-memory architecture [15,37–39]. Furthermore, the high specific surface area of 2D-TMDs results in the distinct absorption and scattering characteristics of radiation energy, which mitigates radiation damage within the material [40–46]. Consequently, 2D-TMDs exhibit unique advantages in radiation resistance compared with conventional 3D materials [47,48], which aligns well with common FPGA application scenarios, including aerospace applications [49].

In this work, we have successfully fabricated an FPGA based on 2D-TMDs with ∼4000 FETs by using a silicon-compatible top-gate (TG) process and *in situ*-grown MoS_2_. We choose MoS_2_ as the channel material because of its proper and adjustable bandgap, high mobility and on/off ratio, excellent stability and potential for integration. The circuit incorporates nine configurable logic blocks (CLBs) routing resources and a memory array. Each CLB consists of a three-input look-up table (LUT3), an edge-triggered D flip-flop (DFF) and a two-input multiplexer (MUX2). The routing resources encompass 96 4-input multiplexers (MUX4), with a routing channel width of 4, and a unidirectional single-length wire architecture. By configuring the configuration bits within the memory array, we have successfully implemented a variety of logic functions, thereby validating its programmability. Furthermore, we have developed a 2D memory array fabrication process compatible with the 2D logic circuit integration process. Each memory cell utilizes only two FETs, which is more compact than the traditional 6T-SRAM-based FPGA memory. Besides, our circuit can withstand a gamma radiation dose of 10 Mrad, which is a significant advantage over silicon-based circuits.

## 2D FPGA STRUCTURE

**Figure 1.**
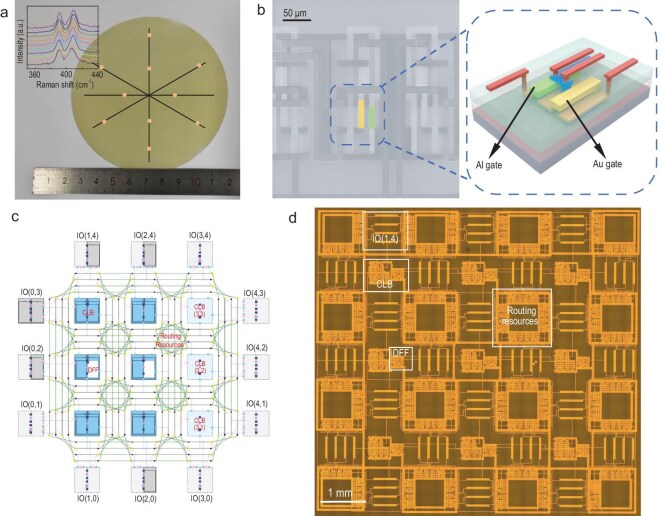
FPGA based on 2D-TMDs. (a) Photograph of a 4-inch MoS_2_ wafer and Raman spectra of 10 random locations on continuous wafer-scale MoS_2_ films. (b) Localized SEM image of the 2D FPGA unit (left) and 3D structural schematic of E-D-type NMOS logic (right). (c) Logical circuit design diagram of the 2D FPGA. (d) Optical microscope image of the 2D FPGA. I/O, input/output.

We demonstrate a complementary metal oxide semiconductor (CMOS)-compatible FPGA circuit architecture for 2D-TMDs. The circuit logic employs enhancement-depletion (E-D)-type N-metal-oxide-semiconductor (NMOS) logic (see Fig. S1), while a 2T0C (2-Transistor, 0-Capacitor) structure is utilized for the memory array. Figure 1a presents a 4-inch single-layer MoS_2_ wafer grown on a sapphire substrate, along with Raman spectra acquired across its surface. The Raman spectra, displaying 10 curves, reveals that the $E_{2{\mathrm{g}}}^1$ and ${A}_{1{\mathrm{g}}}$ peaks are consistently positioned, confirming excellent film uniformity. Figure 1b shows a partial scanning electron microscopy (SEM) image and a 3D schematic of the 2D FPGA logic circuit. The entire circuit features a three-layer interconnected structure (for detailed architecture, see Fig. S2), with metal lines and vias clearly discernible in the SEM image.

Figure 1c and d illustrate the design layout and optical micrograph of the 2D FPGA logic circuit, respectively. The 2D FPGA was designed by using Verilog-to-Routing (VTR) [50]—an open-source framework for FPGA architecture and computer-aided design (CAD) research and development.

In Fig. 1c, each central blue square represents a CLB unit, while the dashed blue squares indicate potential idle or redundant resources within the configuration. The 12 gray squares surrounding the CLBs are the input and output (I/O) pads of the FPGA, with the dashed portions denoting potentially idle or redundant I/O pads. Solid black arrows connecting blue and gray squares depict the routing resources of the FPGA, with arrows indicating the signal transmission direction. Green lines between blue and gray squares illustrate the interconnection of the routing resources, which consist of 96 MUX4s in total. The overall logic circuit consists of nine CLBs, each containing a LUT3, a DFF and a MUX2. The routing channel width is set to 4 and a unidirectional single-length wire architecture is utilized. In Fig. 1d, the key components are highlighted in the corresponding area. The circuit area measures 10 mm × 10 mm, while the total size of the CVD-grown MoS_2_ substrate is 15 mm × 15 mm. The entire circuit has ∼4000 FETs and needs 290 configuration bits, fitting well within a memory array of 20 × 15. This is because each LUT3 has 8 configuration bits and 81 FETs, each MUX2 has 1 configuration bit and 9 FETs, each MUX4 has 2 configuration bits and 27 FETs, and each DFF has 18 FETs but no configuration bits.

### From FETs to CLB

**Figure 2.**
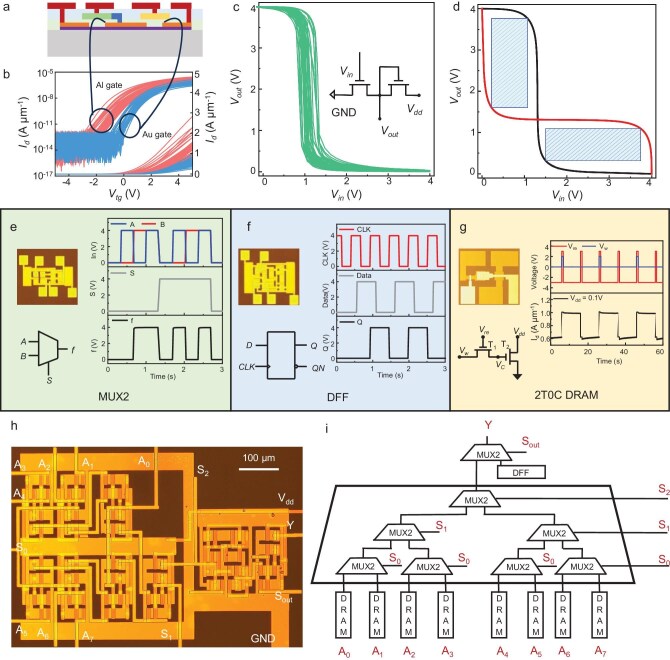
Characterization of FETs and logic. (a) Cross-sectional schematic diagram of the inverter in the circuit. (b) Transfer characteristic curves of FETs corresponding to two types of metals. (c) Transfer characteristic curves of the inverter and structural schematic diagram of a single E-D-type NMOS inverter. (d) Noise margins of the inverter. (e–g) Performance demonstrations of three circuit modules: (e) MUX2, (f) DFF and (g) 2T0C DRAM. (h) Optical image and (i) structural schematic diagram of CLB.

To fabricate the 2D FPGA, a complete fabrication process flow was developed in our previous research [8,24,51] (detailed steps are presented in the Methods section and Figs S3 and S4). For the inverters, which are the fundamental building blocks of logic circuits, the realization of their high performance, excellent stability and maintaining signal integrity in multistage cascading have become key issues that need to be addressed in the field of 2D-material ICs. Figure 2a presents a cross-sectional schematic of the inverter implemented in the logic circuits. Figure 2b illustrates the modulation of the transistor threshold voltage achieved by using two distinct TG metals. High uniformity is achieved through meticulous process control and structural optimization. The two curves correspond to the transfer characteristics of the Au-gated FETs and the Al-gated FETs. As depicted, both transistor types exhibit n-type behavior. The Au-gated FET demonstrates a positive threshold voltage and operates as a typical enhancement-mode FET. In contrast, the Al-gated transistor exhibits a threshold voltage that is negatively shifted by ∼1 V relative to the Au-gated FET. All FETs have channel dimensions of 30 μm in width and 10 μm in length. Detailed statistical analyses of the transistor characteristics, including the threshold voltage and assessments of transistor uniformity, are provided in Fig. S5.

Figure 2c presents the characteristic curves and structure of the E-D-type NMOS inverters utilized in this work. By leveraging the threshold voltage modulation of different TG FETs, we realize 2D inverters with high uniformity and yield. Figure 2d illustrates the noise margin and gain of the E-D-type NMOS inverter. The switching threshold of the inverter is ∼1 V at a *V*_dd_ (Voltage Drain Drain) of 4 V, demonstrating a favorable noise margin with a Noise Margin High (NMH) of 2.7 V and a Noise Margin Low (NML) of 1.1 V. Based on the stable process and high yield of uniform FETs and inverters, the fundamental modules for the FPGA are constructed. Figure 2e–g show the optical micrographs, structural schematics and test results for several key building blocks. Figure 2e depicts the MUX2, which consists of nine FETs and needs three signals (input signals *A* and *B*, and a select signal *S*). At a *V*_dd_ of 4 V, when *S* is low (0 V), output *f* matches input *B*. When *S* is high (4 V), output *f* matches input *A*. This confirms the correct logic function of the MUX2. Figure 2f presents the rising-edge-triggered DFF, made up of 18 FETs. There are two signals: input *D* and clock *CLK*. At *V*_dd_ of 4 V, when *CLK* first rises from 0 to 4 V, output *Q* matches *D* (high). Even if *D* goes low in between, *Q* stays latched. On the second *CLK* rise, *Q* again follows *D* (low). When *D* goes high in between, *Q* remains latched until the third *CLK* rise, when *Q* once more follows *D* (high).

Figure 2g illustrates the 2T0C DRAM memory cell. Each cell has two FETs, *T*_1_ and *T*_2_, with *T*_2_’s gate connected to *T*_1_’s source. The metal oxide semiconductor (MOS) capacitance of *T*_2_ serves as the storage capacitor. Writing is achieved by applying *V*_re_ (Voltage Read) (–3 to 3 V, 0.8-s pulse) to *T*_1_’s gate and *V*_w_ (Voltage Write) (0 to 2 V, 1-s pulse) to *T*_1_’s drain. *V*_w_ remains high when *V*_re_ is high to fully charge the capacitor. As the gate potential of *T*_2_ is raised, the reading of the stored information can be simply achieved by applying *V*_dd_ across the source and drain of *T*_2_ and measuring the current. To erase the stored information, *V*_re_ is applied to *T*_1_’s gate and *V*_w_ = 0 V is applied to *T*_1_’s drain, discharging the stored charge through the drain of *T*_1_. After each write operation, *T*_2_ retains enough charge to enable a source–drain current of ≤1 μA/μm at *V*_ds_ (Voltage Drain Source) = 0.1 V, which decreases gradually due to charge leakage. Within 10 s of retention, the current drops by 5%, indicating that the cell structure retains data effectively for 10 s, which is sufficient for the FPGA configuration’s code storage.

With all the fundamental modules for the FPGA, we finally build up the CLB. The optical image and structural schematic diagram of the CLB are shown in Fig. 2h and i. As shown in Fig. 2i, one CLB block comprises eight MUX2s, one DFF and eight DRAMs. The functions of the CLB will be elaborated on in the subsequent section.

### Demonstrations of 2D FPGA

**Figure 3.**
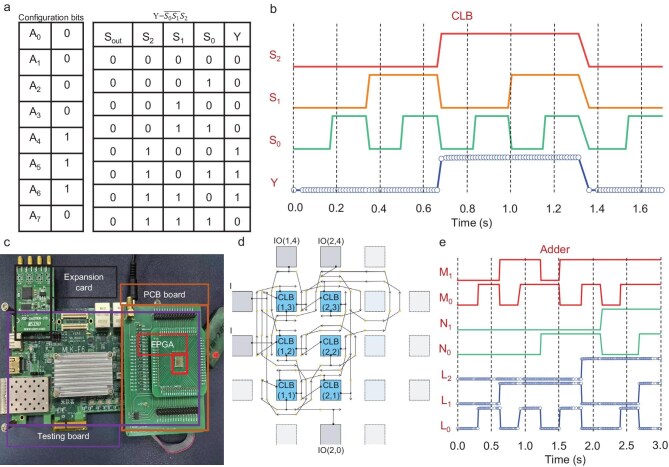
Demonstrations of 2D FPGA. (a) Truth table of the CLB. (b) CLB test results. (c) Testing platform. (d and e) Reconfigurability demonstration of the 2D FPGA: (d) adder configurations of the 2D FPGA to operate as an adder and (d) sequence diagram of the adder.

From FETs to CLB, all basic units have been fabricated by using 2D-TMDs. The final step is to integrate these units but, first, the functionality of the CLB must be demonstrated. The CLB, shown in Fig. 2i, consists of eight configuration bits (*A*_0_ to *A*_7_, each corresponding to one DRAM), three inputs (*S*_0_ to *S*_2_), one *S*_out_ and a single output *Y*. By configuring the configuration bits within the CLB, various logic gates can be implemented. Figure 3a shows the truth table for the logic function implemented by the CLB through the configuration of configuration bits *A*_0_ to *A*_7_. Figure 3b presents the experimental test results, with the timing diagram from the measurements closely matching the truth table, confirming the correct function of the CLB.

After constructing the fundamental components of the 2D FPGA, we validated its programmability by encoding the designed circuit. Figure 3c illustrates the test platform for evaluating complex logic circuits, which includes a ZYNQ MLK-F6-CZ02-7020 FPGA development board, a DAQ-7606 ADC FEP expansion card, a printed circuit board (PCB) for mounting the test sample, and the Vivado 2021.1 FPGA and adaptive SoC design tools software suite. The testing procedure involves the development board generating signals configured by Verilog code, transmitting them to the PCB, routing them to the test sample and collecting output signals for feedback into the test software.

Figure 3d presents the 2D FPGA configuration for a two-bit adder, with the timing diagram results shown in Fig. 3e. This adder employs one LUT2 and two LUT4 units, constructible from two LUT3 and one MUX2 unit each (Fig. S6), with other units as redundant resources. Through different encoding schemes, we also demonstrated a two-bit multiplier and a four-bit counter by using the same FPGA circuit, with their timing diagrams as shown in Fig. S7. The multiplier requires one LUT2 and three LUT3 units, and the counter needs one LUT1, one LUT2, one LUT3, one LUT4 and four DFF units, all contained within the 2D FPGA. Without changing the basic logic units, we successfully implemented these three functions by using the same 2D FPGA circuit, proving its reconfigurability.

### Irradiation test of 2D FPGA

After demonstrating the 2D FPGA, we move to expand its application. Aerospace is a scenario that demands customized hardware embedded in a large system. This kind of scenario is suitable for FPGAs due to their programmability and versatility. But aerospace is a radiation-intense environment in which electronic devices may malfunction. To demonstrate the potential application of the 2D FPGA in aerospace, we applied gamma-ray tests—a standard method for assessing the radiation resistance of electronic devices [52]. Given their application scenarios, we exposed our fabricated FPGA circuits to a total ionizing dose of 10 Mrad of gamma-ray irradiation. By comparing their performance before and after irradiation across different levels from individual FETs to core logic modules, we can accurately evaluate the radiation resistance of FPGAs and identify potential issues, providing valuable data support for enhancing their reliability in radiation environments.

**Figure 4.**
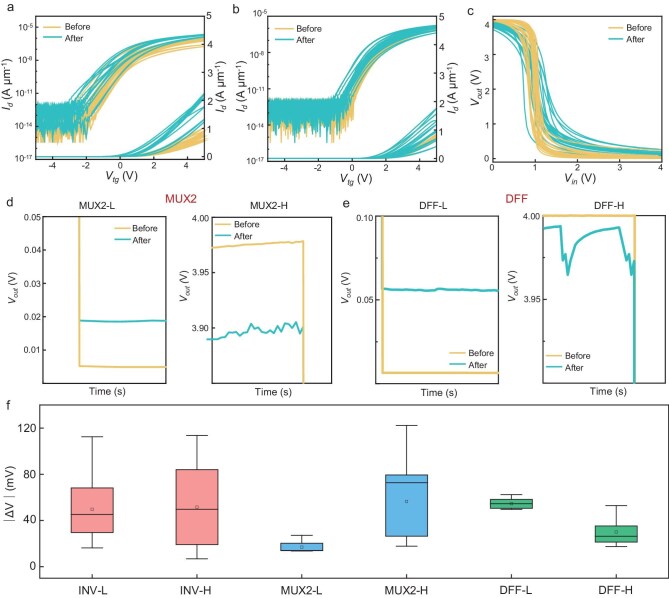
Irradiation test of 2D FPGA. (a and b) Changes in performance of FETs before and after irradiation exposure with gamma rays at a dose of 10 Mrad: (a) Al-gated FETs and (b) Au-gated FETs. (c) Changes in performance of inverters before and after irradiation. (d and e) Changes in performance of logic circuits before and after irradiation: (d) MUX2, (e) DFF and (f) summary of inverters, MUX2 and DFF before and after irradiation in low state and high state.

Figure 4a and b show the changes in transistor performance before and after irradiation. Both transistor types, with the two TG metals, exhibit a slight increase in off-state current and a minor negative shift in threshold voltage. Figure 4c presents the changes in inverter performance. At a *V*_dd_ of 4 V, post-irradiation, some inverters show minor degradation: the high-level output voltage decreases from 4 to ∼3.9 V and the low-level output voltage increases from 0 to ∼0.1 V. The gain and uniformity of the inverters also degrade slightly.

Figure 4d and e illustrate the functionality of the core logic devices MUX2 and DFF, before and after irradiation. The logic timing diagrams show that these units remain functional, though with minor voltage degradation. For the MUX2, the logic low-state voltage rises from ∼0.005 to ∼0.02 V and the high-state voltage drops from ∼3.97 to ∼3.90 V, with noticeable fluctuations. For the DFF, the low-state voltage increases from ∼0.01 to ∼0.05 V and the high-state voltage decreases from ∼4 to ∼3.97 V, also with fluctuations. The above results are summarized in Fig. 4f.

## CONCLUSION

In this work, we constructed an FPGA circuit using MoS_2_, with an integration process compatible with a 2D-TMD-based memory. We validated the feasibility of implementing FPGA functionalities with 2D-TMDs through experimental verification of modules such as the MUX2, DFF, DRAM and CLBs. Using the designed 2D FPGA circuit, we successfully realized various functional circuits, including adders, multipliers and counters.

Our findings show that the circuits, from individual FETs to core logic units, remain functional after being exposed to 10 Mrad of gamma-ray radiation, with minimal impact on performance. Compared with silicon-based and other 2D circuits, ours demonstrate significant advantages, reducing the need for extra protective designs and shielding layers.

This work advances the exploration of large-scale circuits by using 2D-TMD-based TG technology, showing advancements in transistor count and operating voltage. The results hold positive implications for applying 2D-TMDs in large-scale digital circuits.

## METHODS

### Wafer-scale MoS_2_ synthesis

The 4-inch MoS_2_ film growth (on sapphire substrate) underwent an O_2_ plasma treatment to ensure cleanliness and optimal uniformity for film growth. To improve the uniformity of the obtained MoS_2_ film, a high-quality amorphous Al_2_O_3_ thin film with a thickness of 20 nm was deposited as a growth promotion layer by using a thermal atomic layer deposition (ALD) tool (MNT-S300Oz-L4). A sponge-like mixer made of MoO_3_/GO with dimensions of 12 × 12 × 1 mm³ was placed directly below the inverted growth substrate in a quartz tube furnace with a diameter of 200 mm. Pure sulfur sources were positioned symmetrically at both ends of the quartz tube. The growth chamber was evacuated to a pressure of <1 Pa and then purged with argon gas to ensure a contamination-free environment. The MoO_3_/GO sponge and sulfur precursors were heated to 700°C and 220°C, respectively, under a consistent internal chamber vacuum of 0.02 Pa. The growth process lasted for 15 min in the static growth phase.

### Fabrication process of MoS_2_ FETs and circuits

The MoS_2_ FETs and circuits were fabricated on the wafer-scale MoS_2_ film on the sapphire substrate. The contact electrodes were patterned by using laser direct writing technology (Micro-Writer ML3) and subsequently deposited by using E-beam evaporation. O_2_ plasma etching was performed to define a MoS_2_-channel region. A seeding layer was deposited by using E-beam evaporation and subsequently annealed in an oxygen atmosphere. Then, an HfO_2_ layer was grown by using ALD as a high-k dielectric layer. Another lithography/lift-off/deposition process was utilized to form the TG metal layer and metal interconnects. The isolated layer of SiO_2_ was deposited by using physical vapor deposition. More fabrication details can be found in the Supplementary information.

### Electrical measurement

The electrical properties of the MoS_2_ FETs and basic circuits were investigated in a probe station connected to an Agilent B1500A semiconductor analyser with eight source-measure units. To investigate the dynamic response of the circuit, an Agilent 81110A arbitrary-waveform generator was used to input signals, while a RIGOL DS1104 digital oscilloscope and an Agilent B1500A semiconductor analyser captured the output signal. More complex circuits were tested in the unique test platform that consisted of a ZYNQ MLK-F6-CZ02-7020 FPGA development board, a DAQ-7606 ADC FEP expansion card, a PCB board for mounting the bonded test sample, a functional test PCB board, and the associated Vivado 2021.1 FPGA and adaptive SoC design tools software suite.

### Design of 2D FPGA

The 2D FPGA circuits were designed by using the VTR project—an open-source framework for conducting FPGA architecture and CAD research and development. The VTR design flow takes as the input a Verilog description of a digital circuit and a description of the target FPGA architecture. The version of VTR was VTR 8.0 release, which is operated on CentOS Linux release 7.9.2009.

### Irradiation experiments

Irradiation experiments were conducted in an ambient environment by using a ^60^Co γ-ray irradiation test instrument; 10 Mrad γ-rays were radiated at a dose rate of ∼200 krad·h^−1^.

## Supplementary Material

nwaf458_Supplemental_File
