# Supplementary material for: A field-programmable gate array based on wafer-scale 2D semiconductor
Source: Natl Sci Rev. 2025 Oct 31;12(12):nwaf458. doi: 10.1093/nsr/nwaf458 (PMC12721377; doi:10.1093/nsr/nwaf458)
Supplement: nwaf458_Supplemental_File [file nwaf458_supplemental_file.docx]

**Supplementary Information**

**A field-programmable gate array based on wafer-scale two-dimensional semiconductor**

Qicheng Sun^1, a^, Mingrui Ao^1, a^, Xiangqi Dong^1, a^, Kaichuang Shi^1^, Saifei Gou^1^, Yuxuan Zhu^1^, Zhejia Zhang^1^, Jinshu Zhang^1^, Yan Hu^1^, Zhengjie Sun, Xinyu Chen^1^*, Lingli Wang^1^*, Wenzhong Bao^1,2,3^*, Peng Zhou^1,2^*

^1^ State Key Laboratory of Integrated Chips and Systems, College of Integrated Circuits and Micro-Nano Electronics, Fudan University, Shanghai, 200433, China

^2^ Shaoxin Laboratory, Shaoxing, 312000, Zhejiang, China

^3^ Shanghai AtomIC Technology, Shanghai, 201318, China

^a^ These authors contributed equally to this work.

*Corresponding author. Email: [baowz@fudan.edu.cn](mailto:baowz@fudan.edu.cn,); llwang@fudan.edu.cn; 18112020008@fudan.edu.cn; pengzhou@fudan.edu.cn

**
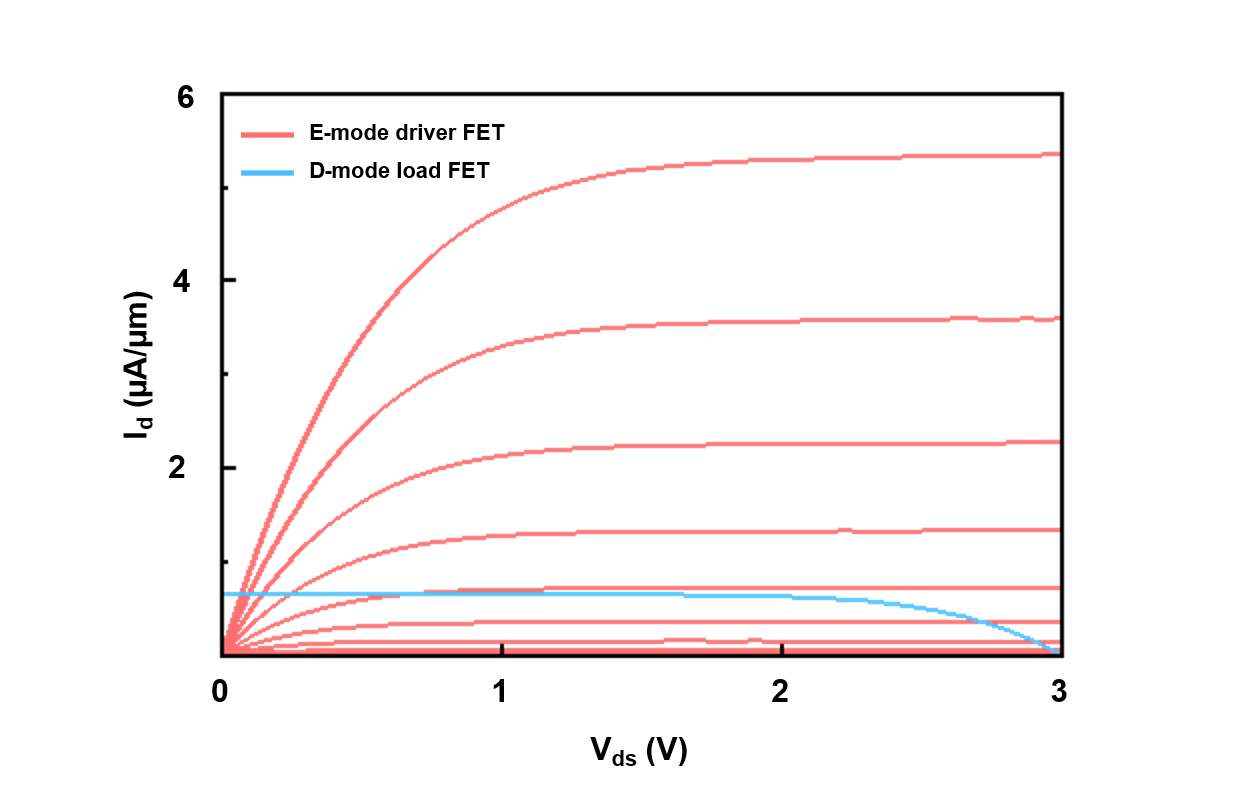
**

**Figure. S1. Output characteristics matching of load and driver FETs for E-D type NMOS inverter.**

**
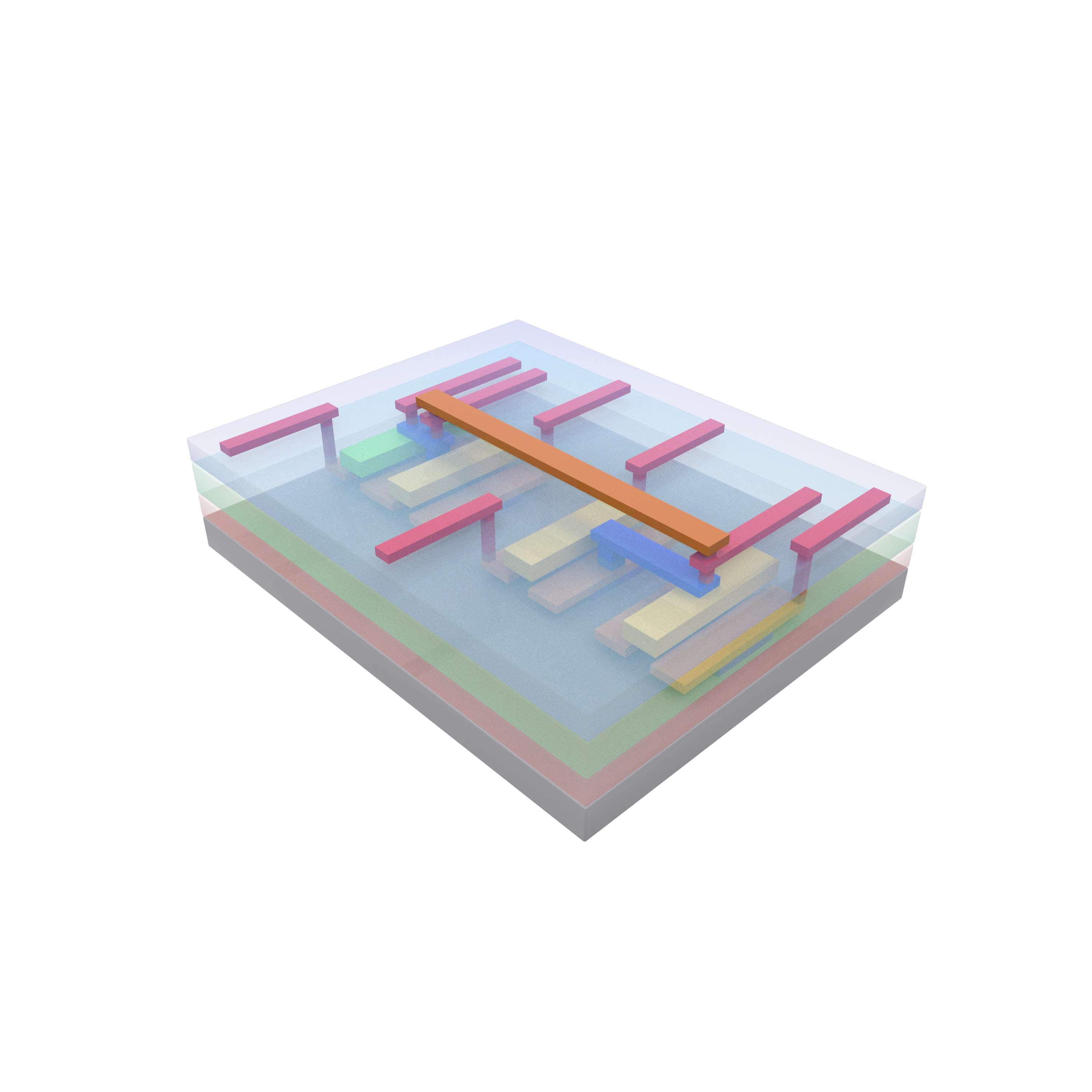
**

**Figure. S2. 3D structure of 2D FPGA.**

**
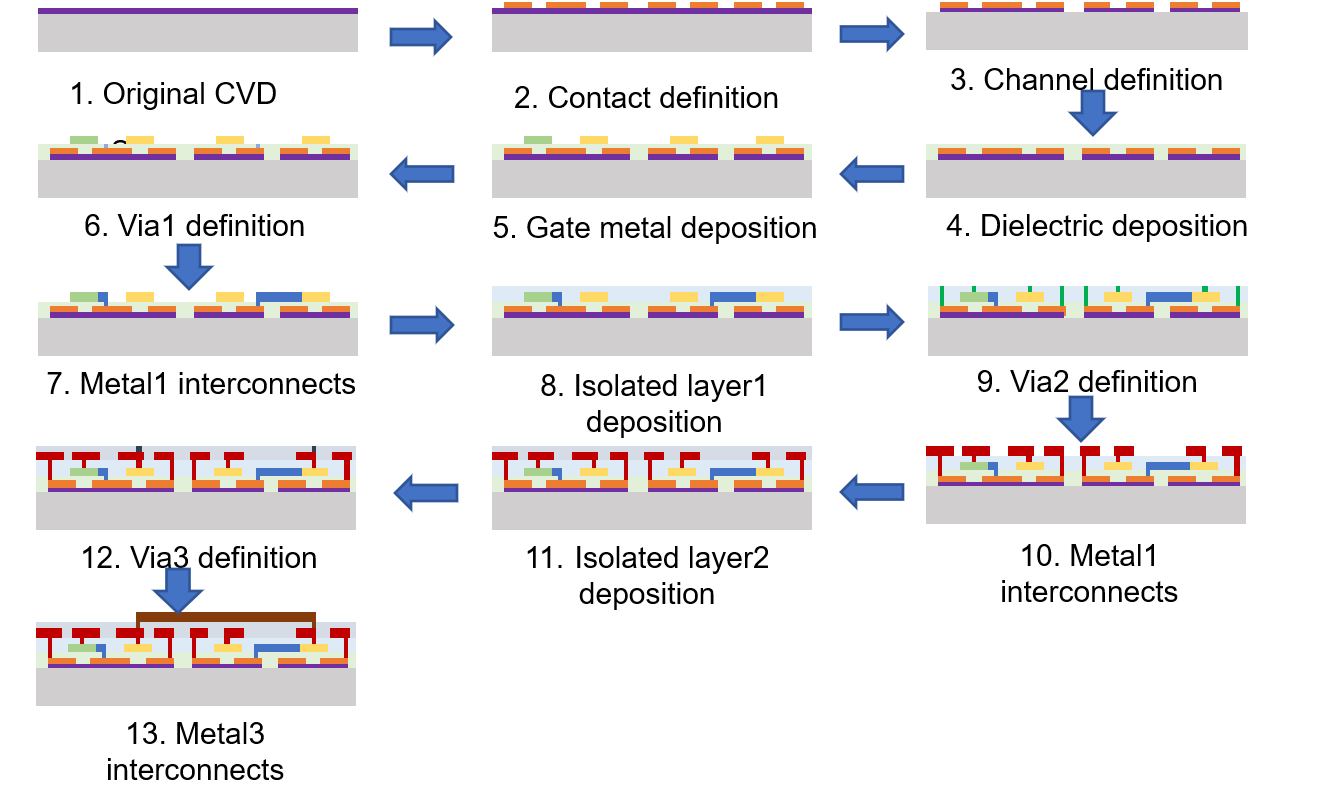
**

**Figure. S3. Fabrication process flow for 2D FPGA (sectional view).**


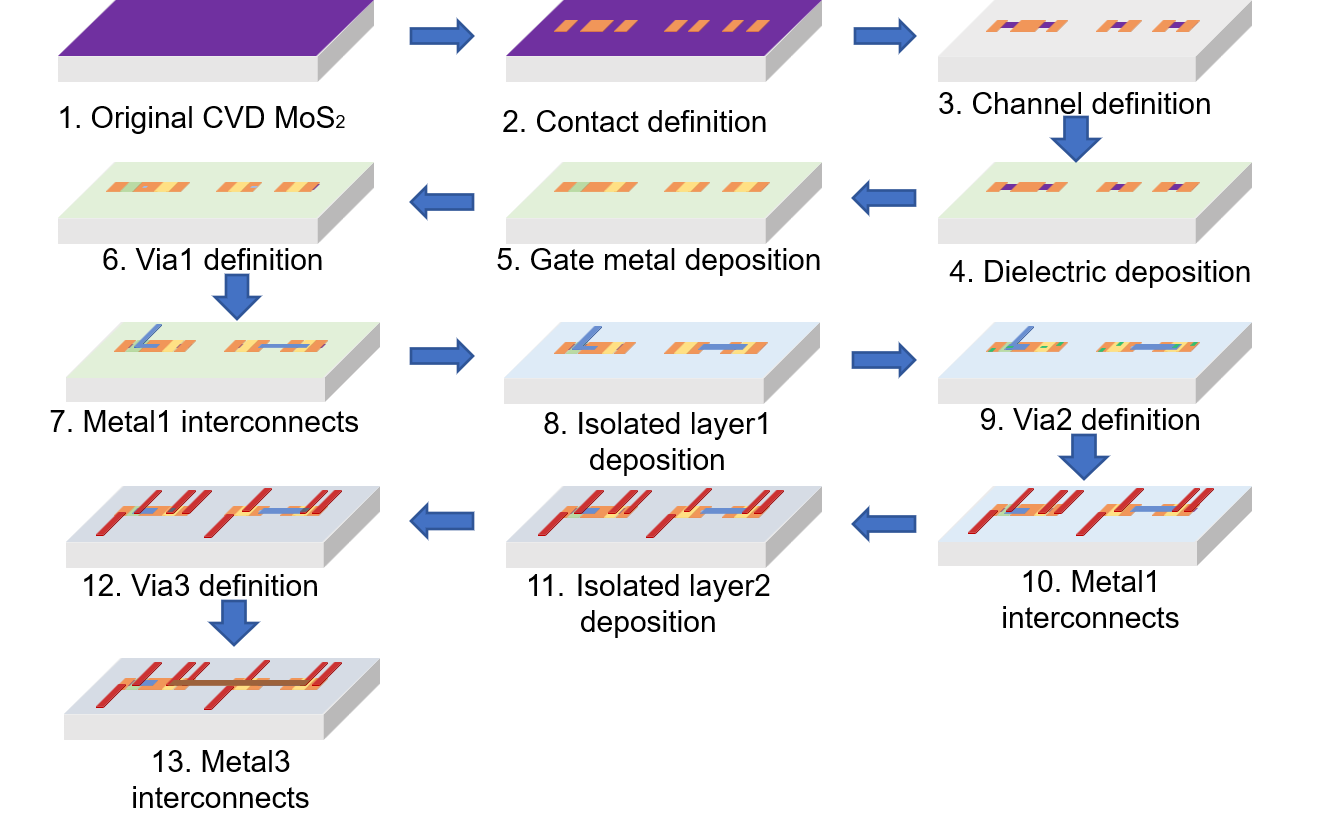


**Figure. S4. Fabrication process flow for 2D FPGA (vertical view).**


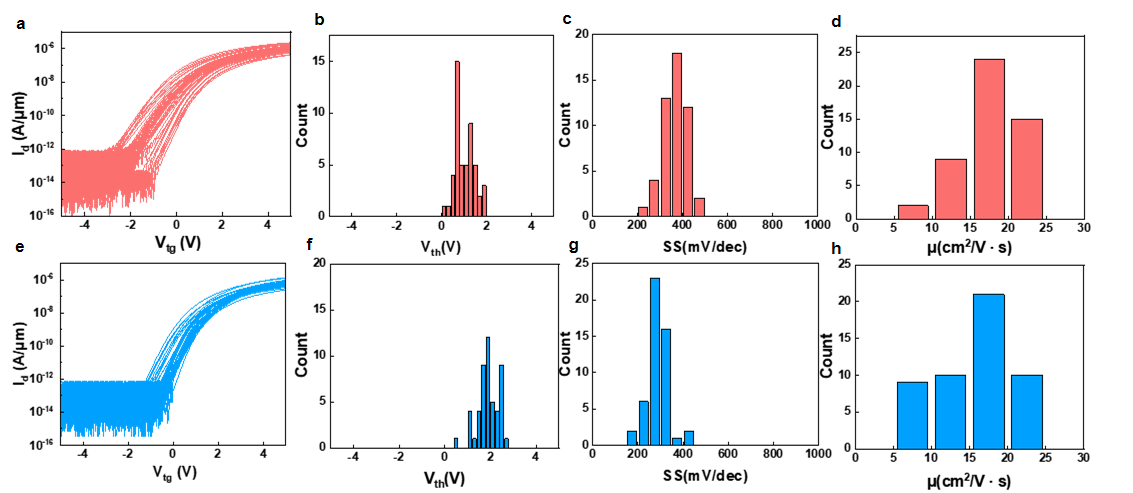


**Figure. S5. Performance of 100 FETs with 50 Al-gated and 50 Au-gated.** Transfer curves (a), Vth (b), SS(c), and mobility (d) of 50 Al-gated FETs. Transfer curves (e), Vth (f), SS(g), and mobility (h) of 50 Au-gated FETs.


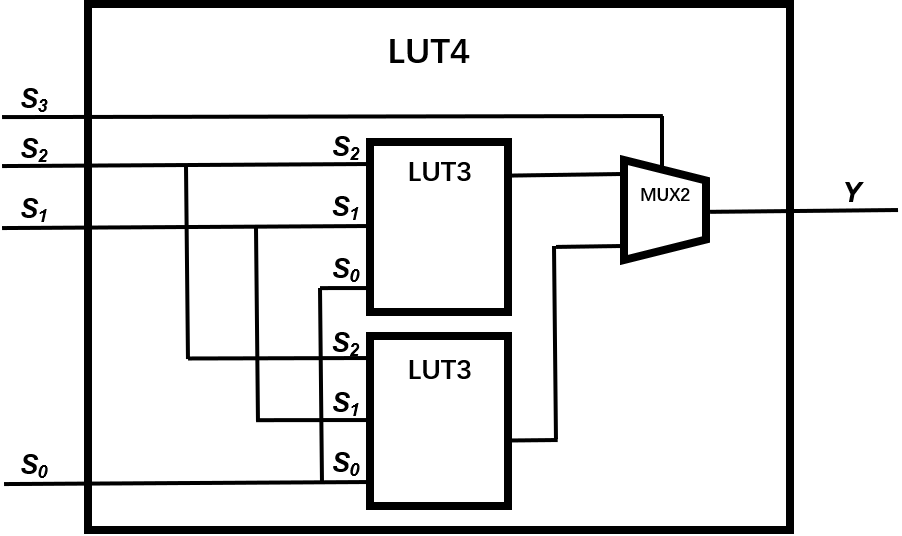


**Figure. S6. One LUT4 consists of one MUX2 and two LUT3.**


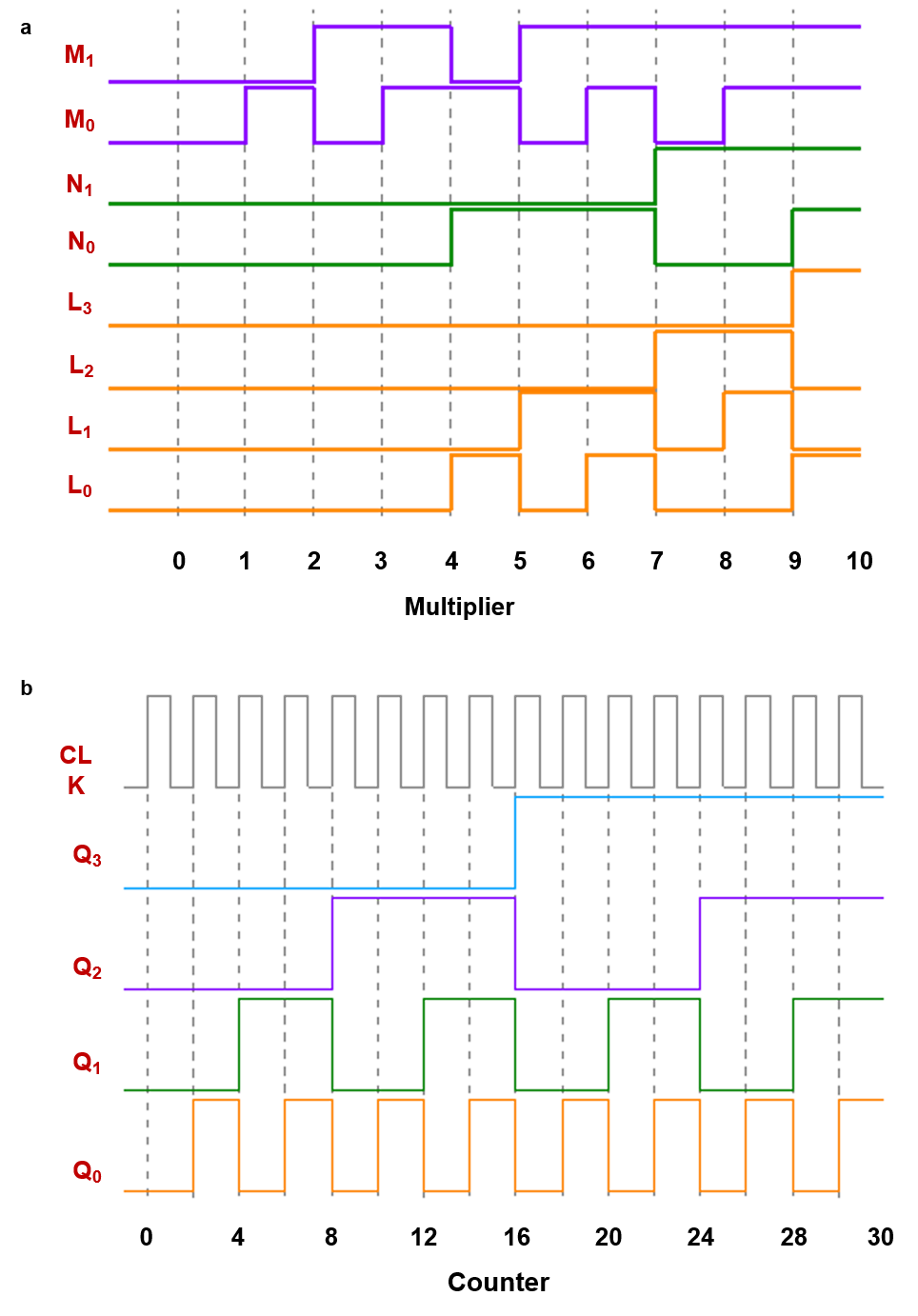


**Figure S7.** Simulations of functional circuits based on 2D FPGA. (a) Multiplier; (b) Counter.


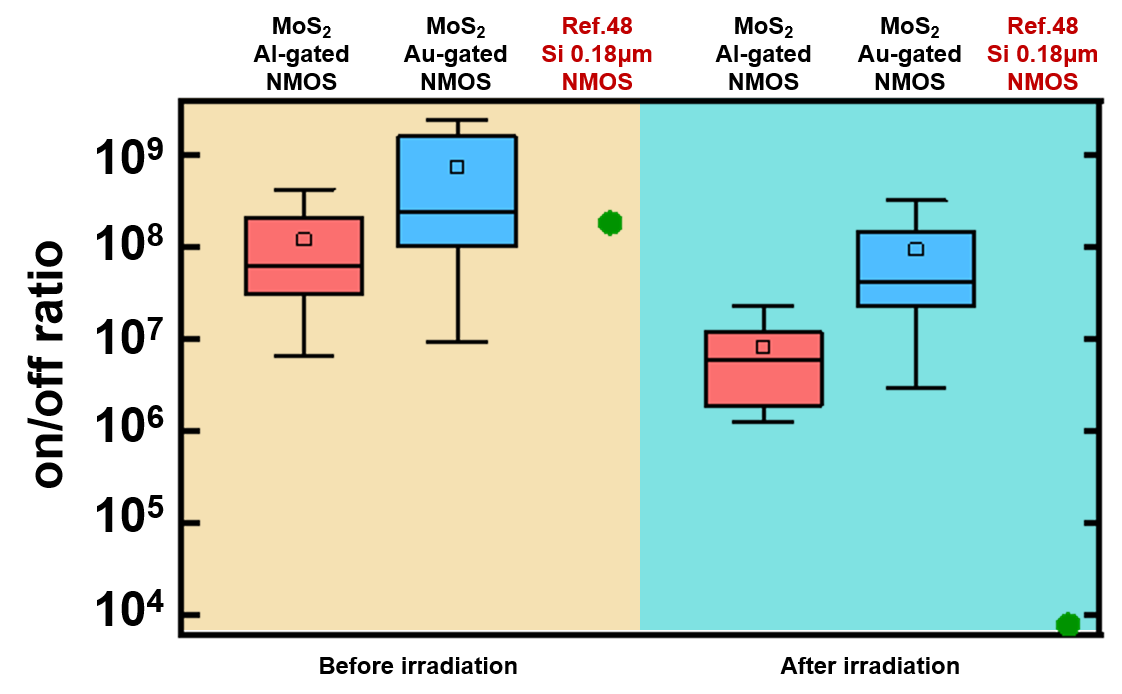


**Figure S8.** Benchmark of irradiation of 2D-TMDs FETs (10Mrad) and Si-0.18μm NMOS-FETs (1Mrad).
